# Editorial for the Special Issue: Recent Advances in Adipokines—2nd Edition

**DOI:** 10.3390/biomedicines13092293

**Published:** 2025-09-18

**Authors:** Christa Buechler

**Affiliations:** Department of Internal Medicine I, University Hospital Regensburg, 93053 Regensburg, Germany; christa.buechler@klinik.uni-regensburg.de

Adipokines are a steadily growing group of bioactive proteins that have mostly been studied in relation to obesity and obesity-associated metabolic diseases [1,2]. Of the more than 500 adipokines identified to date, the majority are linked to overweight or obesity [3,4]. Obese individuals have higher serum levels of certain adipokines, such as leptin and chemerin, and lower levels of adiponectin, omentin, and others [3,4,5]. Composition of the diet can modulate adipokine levels [6,7], and the effect of short-chain fatty acids, which are produced when microorganisms ferment indigestible carbohydrates and/or proteins, and of branched-chain fatty acids, which are produced when proteins are fermented, has been analyzed. Individual fatty acids affected the adipokine profile of 3T3-L1 adipocytes in inflammation under normoxic and hypoxic experimental conditions, showing that type of diet may modulate serum adipokine levels [8]. There is also evidence that membrane fatty acid composition is related to serum adiponectin [9]. The fatty acid composition of red blood cell membranes is thought to be a reliable indicator for long-term fatty acid intake [10]. Red blood cell membrane fatty acid profiles in obese individuals revealed a reduction in total omega-3 fatty acids, primarily due to lower levels of docosahexaenoic acid. Conversely, there was an increase in total omega-6 fatty acids, primarily due to higher levels of dihomo-γ-linolenic acid and arachidonic acid [9]. Higher levels of the omega-6 fatty acid dihomo-γ-linolenic acid seem to be related to low adiponectin in obese individuals, as there was a negative correlation between this fatty acid and adiponectin [9].

White adipose tissues that have been studied the most are those found in the subcutaneous and visceral regions [11,12]. Unlike white fat, brown adipose tissue secretes a unique set of hormones known as ‘brown adipokines’ or ‘batokines’, exerting beneficial effects [13,14,15]. Brown fat-derived proteins such as fibroblast growth factor 21 have to be studied in detail, as they may emerge as potent regulators of cardiovascular function [13]. White adipose tissue produces more adiponectin and fibroblast growth factor 21 through calorie restriction or fasting. These hormones play a variety of roles in different tissues, regulating metabolic diseases, delaying aging, and increasing longevity. Notably, centenarian research has revealed reduced adiposity and elevated adiponectin levels, indicating adiponectin to be associated with healthy aging [16].

Although females have higher systemic adiponectin levels than males [17], research on sex-specific associations is limited. Sex and metabolic syndrome are strong determinants of adiponectin–serum lipoprotein associations [18]. The network involving adiponectin and other molecules that regulate lipoprotein metabolism was found to be primarily active in healthy males and females with metabolic syndrome [18]. This study revealed that analyzing the sex-specific associations and functions of adipokines is recommended.

Adipokines play a vital role in controlling metabolic processes and overall energy balance by mediating communication between adipose tissue and other organs, including the brain, intestines and cardiovascular system [19]. A number of metabolic disorders, including diabetes and cardiovascular disorders, have been linked to the dysregulation of adipokines [19]. Maintaining cardiometabolic health depends on the communication between different tissues. Therefore, understanding how adipokines work can inform the development of treatment strategies for cardiovascular disease and diabetes [19].

As mentioned above, most research on adipokines focuses on metabolic diseases and obesity. It is also worth noting that obesity can exacerbate other inflammatory conditions, such as osteoarthritis, a progressive condition affecting the articular joints [20,21,22]. Leptin and adiponectin are adipokines that have been linked to cartilage degradation and inflammatory processes in joint tissues [23].

The contribution of apelin-related peptides to the development of atherosclerosis is still unclear. However, numerous studies suggest that apelin or Elabela activity may benefit patients with coronary artery disease due to their vasodilatory effects on coronary arteries and cardioprotective potential [24]. Serum Elabela and apelin-17 levels significantly decreased, and apelin receptor significantly increased in the patients with multivessel coronary artery disease in comparison to the healthy controls. As correlations were observed between serum and epicardial adipose tissue for the Elabela and apelin receptor, it is suggested that epicardial adipose tissue plays a role in disease progression [25].

Thickening and calcification of the aortic valve are hallmarks of fibro-calcific aortic valve disease, a progressive condition that ultimately results in aortic stenosis [26]. While the relationship between adiponectin levels and severe aortic stenosis is unclear, a meta-analysis suggests a possible correlation between elevated leptin levels and this disease. These results highlight the need for further research to clarify the role of adipokines in the pathophysiology of fibro-calcific aortic valve disease, as well as their potential as diagnostic biomarkers [27].

Chemerin is a protein that has been the focus of a lot of research and is produced by both hepatocytes and adipocytes [28]. Hypertension, insulin resistance, inflammation, and cancer are all linked to chemerin [29,30,31]. Most of the chemerin in the blood is inactive, and C-terminal processing produces isoforms with different biological activities [32,33]. Patients with type 2 diabetes and a normal body mass index had higher plasma levels of cleaved chemerin than overweight patients with type 2 diabetes, whereas total plasma chemerin levels were similar in both groups [34]. A further study showed an association of chemerin cleavage with insulin resistance. Here, the proteolytic processing of chemerin was highest in patients with type 2 diabetes and was second highest in those who were insulin resistant, resulting in increased levels of cleaved and degraded chemerin, whose biological roles have still to be clarified [35].

Drugs that improve metabolic health may affect serum adipokine levels directly or as a result of improved health [36]. Sodium–glucose co-transporter-2 inhibitors are now an essential part of the treatment for both type 2 diabetes and heart failure. They enhance endothelial function and delay the fibrosis of the heart muscle, providing cardioprotection [37]. Sodium–glucose co-transporter-2 inhibitors may increase adiponectin levels in the blood, thereby raising insulin sensitivity and promoting weight loss. Currently, studies investigating the associations between these drugs and adipokines are scarce, and further research is required [37].

The function of adipokines in critical illness is also the subject of research [38,39]. Among patients with systemic inflammatory response syndrome or sepsis, females infected with SARS-CoV-2 had lower plasma adiponectin levels than females with other causes of severe disease. This effect was not observed in males [40]. Compared to COVID-19 patients with moderate disease and healthy controls, who showed comparable serum adiponectin levels, cases with severe SARS-CoV-2 infection had significantly lower levels of adiponectin. Serum adiponectin levels of severe COVID-19 cases with vancomycin-resistant enterococcal bloodstream infection were further reduced. A sex-specific analysis was not performed in this cohort, which is a limitation of this study [41]. However, serum adiponectin was increased in critically ill patients with liver disease. There were significant positive associations with measures of liver disease severity, such as bilirubin levels, the Model for End-Stage Liver Disease score, and the Child–Pugh score. In these patients, serum adiponectin was associated with a poor outcome [42]. Serum leptin levels were not related to survival in critically ill patients with liver diseases but were found appropriate to exclude diagnosis of acute liver failure in patients with acute liver dysfunction, which is of clinical relevance [43].

The majority of adipokine receptors lack adequate characterization [44]. Agonists and antagonists of adipokine receptors may be identified as potential therapeutics [3,45]. This has been addressed by a recent study related to the role of adiponectin, which mostly exerts a protective role in metabolism [46,47]. Blocking peripheral adiponectin receptors impaired the cognitive function of mice and increased anxiety-like behaviors [48]. Mice with inhibited peripheral adiponectin receptors also accumulated β-amyloid, suggesting a role in Alzheimer’s disease [48]. Leptin is necessary for maintaining energy balance and significantly impacts brain functions, particularly in the hippocampus, which is crucial for memory and learning. Reduced responsiveness to leptin is a sign of leptin resistance, which occurs in obesity [49] and impairs the function of the hippocampus, thereby exacerbating obesity and cognitive decline [50].

Taking everything into account, the original and review papers in this Special Issue demonstrate the diversity of adipokines and their numerous roles in health and disease. Developments in this area may result in better patient management in terms of diagnosis, prognosis, and the creation of novel treatments.
